# Is ampicillin plus cephalosporins a therapeutic option for Ampicillin-Susceptible *Enterococcus faecium*?

**DOI:** 10.1093/jac/dkaf226

**Published:** 2025-08-06

**Authors:** Paula Bierge, Inmaculada Gómez-Sánchez, Cristina Garcia de la Mària, Miquel Sánchez-Osuna, Silvia Capilla, Antonio Casabella, Mateu Espasa, Carla Novais, Jose M Miró, Oscar Q Pich, Oriol Gasch

**Affiliations:** Laboratori de Recerca en Microbiologia i Malalties Infeccioses, Hospital Universitari Parc Taulí, Institut d’Investigació i Innovació Parc Taulí (I3PT-CERCA), Universitat Autònoma de Barcelona, Sabadell, Spain; Institut de Biotecnologia i Biomedicina, Universitat Autònoma de Barcelona, Cerdanyola del Vallès, Spain; Laboratori de Recerca en Microbiologia i Malalties Infeccioses, Hospital Universitari Parc Taulí, Institut d’Investigació i Innovació Parc Taulí (I3PT-CERCA), Universitat Autònoma de Barcelona, Sabadell, Spain; Institut de Biotecnologia i Biomedicina, Universitat Autònoma de Barcelona, Cerdanyola del Vallès, Spain; Departament de Microbiologia, Hospital Clinic-IDIBAPS, Universitat de Barcelona, Barcelona, Spain; Laboratori de Recerca en Microbiologia i Malalties Infeccioses, Hospital Universitari Parc Taulí, Institut d’Investigació i Innovació Parc Taulí (I3PT-CERCA), Universitat Autònoma de Barcelona, Sabadell, Spain; Institut de Biotecnologia i Biomedicina, Universitat Autònoma de Barcelona, Cerdanyola del Vallès, Spain; Departament de Microbiologia. Laboratoris Clínics-UDIAT, Hospital Universitari Parc Taulí, Institut d’Investigació i Innovació Parc Taulí (I3PT-CERCA), Universitat Autònoma de Barcelona, Sabadell, Spain; Departament de Microbiologia. Laboratoris Clínics-UDIAT, Hospital Universitari Parc Taulí, Institut d’Investigació i Innovació Parc Taulí (I3PT-CERCA), Universitat Autònoma de Barcelona, Sabadell, Spain; Departament de Microbiologia. Laboratoris Clínics-UDIAT, Hospital Universitari Parc Taulí, Institut d’Investigació i Innovació Parc Taulí (I3PT-CERCA), Universitat Autònoma de Barcelona, Sabadell, Spain; UCIBIO- Unidade de Ciências Biomoleculares Aplicadas, Faculdade de Farmácia, Universidade do Porto, Porto, Portugal; Laboratório Associado i4HB—Instituto para a Saúde e a Bioeconomia, Faculdade de Farmácia, Universidade do Porto, Porto, Portugal; Departament de Microbiologia, Hospital Clinic-IDIBAPS, Universitat de Barcelona, Barcelona, Spain; Laboratori de Recerca en Microbiologia i Malalties Infeccioses, Hospital Universitari Parc Taulí, Institut d’Investigació i Innovació Parc Taulí (I3PT-CERCA), Universitat Autònoma de Barcelona, Sabadell, Spain; Institut de Biotecnologia i Biomedicina, Universitat Autònoma de Barcelona, Cerdanyola del Vallès, Spain; Servei de Malalties Infeccioses, Hospital Universitari Parc Taulí, Institut d’Investigació i Innovació Parc Taulí (I3PT-CERCA), Universitat Autònoma de Barcelona, Sabadell, Spain

## Abstract

**Objectives:**

*Enterococcus faecium*, a significant hospital associated pathogen, poses substantial treatment challenges. While combinations of ampicillin with cephalosporins are first-line therapies to treat *Enterococcus faecalis* high-mortality-rates infections, their efficacy against ampicillin-susceptible *E. faecium* (ASEfm) is less clear. This study evaluates the effectiveness of combining ampicillin with ceftriaxone or ceftaroline against ASEfm strains.

**Methods:**

Ten ASEfm bloodstream isolates from complicated infections were analyzed. Susceptibility to several antibiotics, including ceftriaxone, ceftaroline, and cefotaxime, was determined using standard methods. Time-kill assays were conducted to assess the synergistic and additive effects of ampicillin in combination with ceftriaxone or ceftaroline.

**Results:**

Time-kill studies revealed that ampicillin combined with ceftaroline demonstrated synergistic and/or additive effects in 7 out of 10 strains analyzed. In contrast, ampicillin combined with ceftriaxone showed less pronounced synergy, with only 3 out of 10 strains exhibiting synergistic and/or additive effects.

**Conclusions:**

Our findings indicate that ampicillin combined with ceftaroline, or ceftriaxone provides synergistic activity against some but not all ASEfm clinical isolates. The observed synergy suggests that these combinations could offer a potential therapeutic option for challenging ASEfm infections.

## Introduction

*Enterococcus faecium* has emerged as a major clinical pathogen, posing a considerable challenge in hospital environments, particularly among immunocompromised patients. It is associated with a broad range of healthcare-associated infections, including infective endocarditis, which is frequently difficult to treat. Therapeutic options are increasingly constrained by the organism’s intrinsic resistance to multiple antibiotic classes, further exacerbated by the frequent acquisition of additional resistance mechanisms, such as vancomycin resistance.^1^ As resistance extends even to last-resort agents, the development of novel therapeutic strategies or optimized antibiotic combinations has become an urgent clinical priority.^1,2^

Ampicillin has been the cornerstone to treat infections caused by enterococci susceptible to β-lactam antibiotics due to its efficacy in inhibiting cell wall synthesis. However, *E. faecium* has developed resistance mechanisms that significantly reduce the effectiveness of ampicillin. A key mechanism involves alterations in Penicillin-Binding Proteins (PBPs), specifically Pbp5, which have a reduced affinity for β-lactam antibiotics, further contributing to resistance. Meanwhile, cephalosporins’ effectiveness against *Enterococcus* is limited; thus, they are not used as monotherapy.^3^

β-lactam monotherapy has been associated with a bacteriostatic effect in the aforementioned difficult-to-treat infections, resulting in poor outcomes.^4^ Consequently β-lactams are commonly combined with aminoglycosides to utilize their synergistic effects.^5^ Endocarditis guidelines from the American Heart Association and the European Society of Cardiology recommend the use of ampicillin plus ceftriaxone for *Enterococcus faecalis* endocarditis, showing high success rates and no increased risk of renal impairment associated with aminoglycosides.^6^ Synergism between ampicillin and ceftaroline against *E. faecalis* has also been documented.^7^

However, despite the clinical relevance of *E. faecium*, particularly in hospital-acquired infections, data on the effectiveness of β-lactam combinations in this species are limited. *In vitro* studies with ampicillin-susceptible *Enterococcus faecium* (ASEfm) indicate that ampicillin and ceftriaxone do not consistently demonstrate synergy.^8^ Moreover, there is a clear gap in the literature regarding the efficacy and variability of ampicillin-based combination—particularly with ceftriaxone or ceftaroline—against ASEfm.

This study aims to address the gap in knowledge regarding the efficacy of ampicillin combined with cephalosporins, specifically ceftriaxone or ceftaroline, against ASEfm.

## Material and methods

### Strains and culture conditions

Ten ASEfm bloodstream isolates from complicated bacteraemia and infective endocarditis episodes were identified at Parc Taulí Hospital (2014–2021) using a MALDI-TOF MS instrument (Bruker, Billerica, MA, USA). Patient comorbidities, source of bacteraemia and acquisition, antibiotic therapy and outcomes were recorded. Bacterial cultures were grown in cation-adjusted Mueller-Hinton broth (Sigma Aldrich, St Louis, MO, USA).

### Whole genome sequencing and multilocus sequence typing

*E. faecium* isolates were cultured on Columbia agar plates supplemented with 5% sheep blood (bioMérieux) and incubated at 37°C. Genomic DNA was extracted using the DNeasy Blood & Tissue Kit (Qiagen), and its quality was assessed with a Qubit^®^ 2.0 fluorometer (Thermo Fisher Scientific). DNA libraries were prepared using the Nextera XT DNA Sample Preparation Kit (Illumina), and paired-end whole-genome sequencing was carried out on an Illumina HiSeq 2500 platform at the Centre for Genomic Regulation (CRG, Barcelona). Raw sequencing read quality was assessed using FastQC (https://github.com/s-andrews/FastQC). Reads were pre-processed and filtered with TrimGalore (https://github.com/FelixKrueger/TrimGalore), followed by *de novo* assembly using SPAdes.^9^ The resulting assemblies were evaluated for quality using CheckM2.^10^ Multilocus sequence typing (MLST) was performed using the MLST software (https://github.com/tseemann/mlst), based on the recently published MLST scheme for *E. faecium*.^11^ Novel STs were submitted to PubMLST for future reference.

### Antibiotic susceptibility testing

Antibiotic susceptibility for cephalosporins, ampicillin and vancomycin was determined using the MicroScan system (Dade Behring, West Sacramento, CABD) from 2014 to 2019 and the Phoenix system (BD Phoenix^™^, Franklin Lakes, USA) from 2020 to 2021. Cephalosporins and ampicillin susceptibility was further confirmed using the Etest (BioMérieux, Marcy-l’Étoile, France), following guidelines of the EUCAST.^12^ To validate the MIC values used for the time-kill assays, standard broth microdilution was also performed, and results are provided in Table S1b (available as Supplementary data at *JAC* Online). All tests were performed in triplicate. The results were expressed as the median and the interquartile range (IQR).

### *In vitro* time-kill curves

Time-kill curves (TKCs) were conducted to evaluate the bactericidal and synergistic effects of various combination regimens against ASEfm strains. The antibiotics used were ampicillin (Sigma Aldrich, St Louis, MO, USA), ceftriaxone (Sigma Aldrich, St Louis, MO, USA), and ceftaroline (Pfizer, New York, NY, USA), alone or combined, following the criteria described elsewhere.^8,13^ The regimens included ½ × MIC or 1 × MIC levels of the drugs, with an initial inoculum of 5 × 10^5^ cfu/mL. For ASEfm resistant to ceftriaxone, experiments were performed using 150 mg/L of the antibiotic, approximately its peak serum concentration (C_max_) after a single dose.^14^ Culture aliquots were sampled at 0, 4, and 24 h for each strain and condition and bacterial viability was determined (log_10_ cfu/mL). Quality controls were performed throughout to ensure antibiotic efficacy. Each experiment was conducted in triplicate.

A bactericidal effect was defined as a reduction of at least 3 log_10_ cfu/mL compared with the initial inoculum, while synergistic effects were confirmed by at least a 2 log_10_ cfu/mL reduction at 24 h compared with the most active single agent within the combination. The additive effect was considered when the combination resulted in a reduction of 1 to <2 log_10_ cfu/mL compared with the most active antibiotic. Indifference occurred when the combination showed a change ±1 log_10_ cfu/mL compared with the most active antibiotic.^13^

## Results

### Antibiotic susceptibility

**Figure 1.**
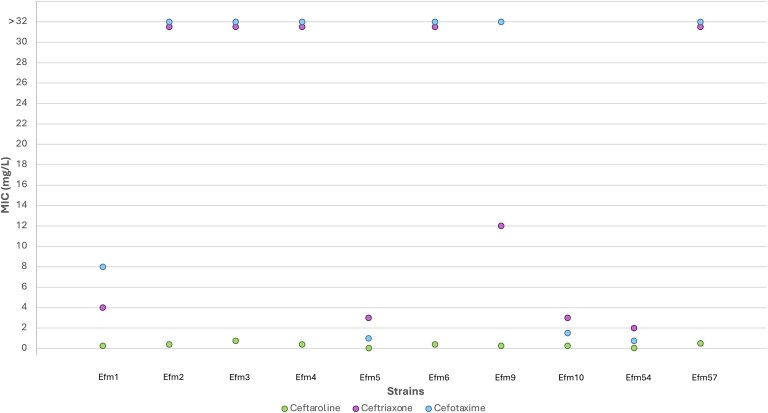
MICs of ceftriaxone, cefotaxime, and ceftaroline against the 10 ASEfm strains.

All the ASEfm isolates under study were susceptible to ampicillin, as detailed in Table S1. Ampicillin displayed the lowest MIC values across all isolates. In contrast, the strains studied revealed variable susceptibility to cephalosporins—ceftaroline, ceftriaxone, and cefotaxime— as shown in Figure 1 and Table S1. Ceftaroline exhibited uniformly low MIC values across all isolates, ranging from 0.25 mg/L to 0.75 mg/L (Median = 0.38 mg/L, IQR 0.2825–0.5 mg/L). However, ceftriaxone (Median = 30 mg/L, IQR 3.25–256 mg/L) and cefotaxime (Median = 32 mg/L, IQR 3.125–32 mg/L) showed more variable results. Isolates Efm1, Efm5, Efm10, and Efm54 had low MIC values (≤8 mg/L), while isolates Efm3, Efm4, Efm6, and Efm57 displayed higher MIC values (>32 mg/L). Notably, isolate Efm54 was the most susceptible to all the antibiotics tested, including ceftaroline (0.064 mg/L), ceftriaxone (1 mg/L), and cefotaxime (0.75 mg/L).

### Sequence type

Genetic variability was observed among the strains, as they were categorized into different STs [Table 1], including both previously identified and novel STs.

**Table 1.** Clinical characteristics of the patients from whom the strains under study were isolated

Strains (isolation)	ST	Age/sex	Comorbidities	Acquisition and source of infection	Antibiotic therapy	Outcome (persistence/mortality)
Efm1 (July 2014)	ST1522	79/M	Hypertension, diabetes mellitus, mild cognitive impairment	Community acquired, Biliary	Amoxicillin-clavulanate	No/no
Efm2 (March 2015)	ST1521	85/F	Pancreatic neoplasm	Healthcare-associated, Biliary	Meropenem	No/no
Efm3 (March 2015)	ST242	66/M	Cholangiocarcinoma	Nosocomial, Biliary	Amoxicillin-clavulanate	No/yes
Efm4 (March 2015)	ST1519	73/M	Head of pancreas neoplasm with metastasis and additional tumour spread	Healthcare-associated, Hepatic abscess	Meropenem	No/no
Efm5 (May 2015)	ST1195	89/F	Total dependence, cerebrovascular accident	Healthcare-associated, Urinary	Levofloxacin + clindamycin	No/no
Efm6 (June 2015)	ST1517	86/F	Alzheimer’s disease	Community-acquired, Urinary	Amoxicillin	No/no
Efm9 (September 2020)	ST1520	46/F	Crohn’s disease	Healthcare-associated, Digestive	Linezolid	No/no
Efm10 (September 2020)	ST40	74/M	Congestive heart failure, cirrhosis, valvulopathy	Nosocomial, Endocarditis	Daptomycin + ceftaroline; daptomycin + imipenem; ampicillin + ceftaroline	No/no
Efm54 (August 2021)	ST1518	86/M	Oropharyngeal neoplasm with chemotherapy	Community acquired, Unknown focus	Teicoplanin; ampicillin	No/no
Efm57 (July 2020)	ST592	64/F	Admission for cholangitis secondary to choledocholithiasis	Healthcare-associated, Biliary	Meropenem; linezolid	No/no

F, female; M, male; ST, Sequence Type.

### *In vitro* time-kill studies

**Figure 2.**
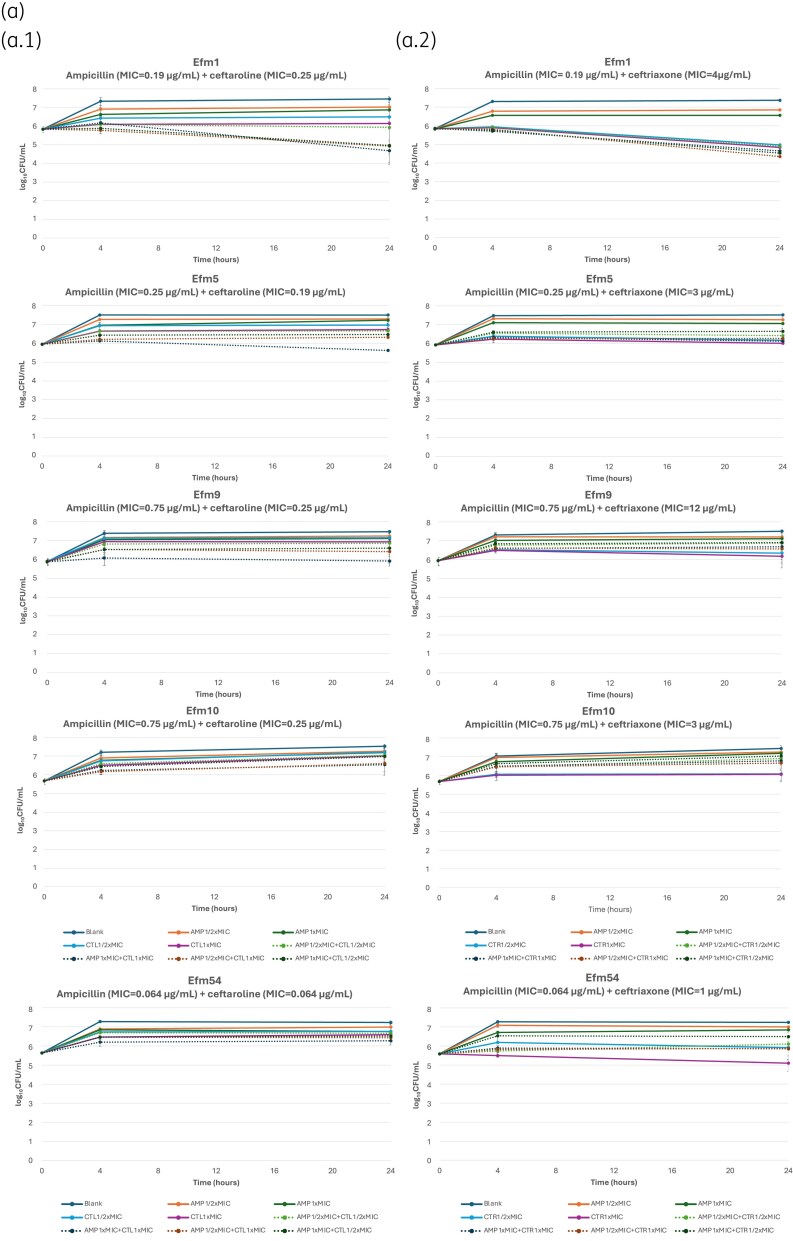

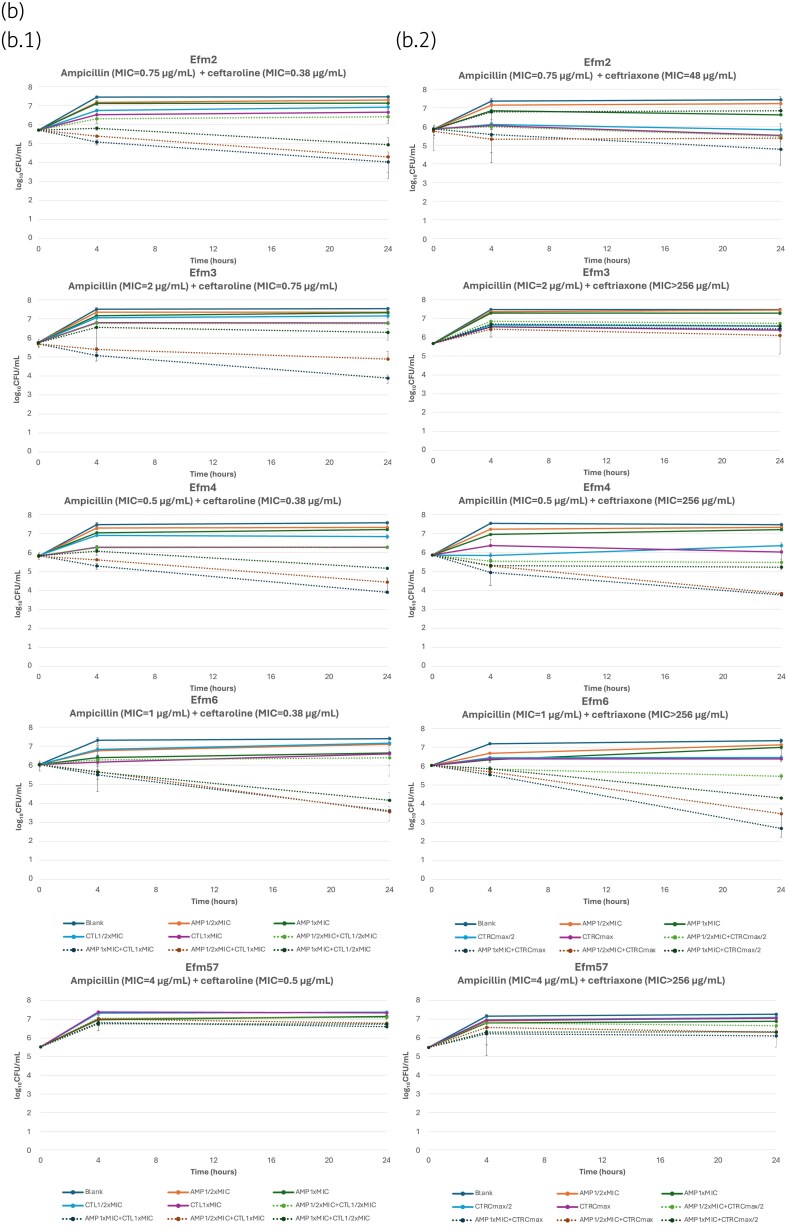
(a) TKCs for combinations of ampicillin (AMP) with ceftaroline (CTL) or ceftriaxone (CTR) against the five AMP-S *E. faecium* strains with low MICs to ceftriaxone. Panel (a.1) depicts the TKCs for the combination of AMP with CTL, while panel (a.2) shows the TKCs for the combination of AMP with CTR. Each curve represents the bacterial count (log10 cfu/mL) over time. (b) TKCs for combinations of AMP with CTL or CTR against the five AMP-S *E. faecium* strains with high MICs to ceftriaxone. Panel (b.1) depicts the TKCs for the combination of AMP with CTL, while panel (b.2) shows the TKCs for the combination of AMP with CTR. Each curve represents the bacterial count (log_10_ cfu/mL) over time.

As expected, monotherapies did not exhibit bactericidal effects, as the experiments were conducted using sublethal antibiotic concentrations. The combination of ampicillin and ceftaroline against ASEfm strains demonstrated varied interactions. Notably, 7 out of 10 strains exhibited either synergistic and/or additive effects [Figure 2a and b, Tables S2 and S3]. Efm6, for instance, showed a marked synergy with a ΔChange of −3.05 log10 cfu/mL at 1xMIC of ceftaroline, indicating substantial inhibitory effect.

The combination of ampicillin and ceftriaxone exhibited less effective interactions compared with the combination of ampicillin and ceftaroline. Specifically, 3 out of 10 strains showed either synergistic and/or additive effects, while the remaining strains display indifferent effects. Notably, Efm6 showed a marked synergy with a ΔChange of −3.68 log10 cfu/mL at 1 × Cmax of ceftriaxone, achieving a bactericidal effect, an outcome not observed in any other strain analyzed. These results suggest that while the combinations of ampicillin with either ceftaroline or ceftriaxone can enhance growth inhibition for certain strains through synergistic and additive interactions, they are generally insufficient for complete bacterial eradication at the concentrations used in our experiments.

## Discussion

This study investigates the impact of combining ampicillin with either ceftriaxone or ceftaroline against clinical ASEfm strains as potential therapeutic strategies for challenging difficult-to-treat ASEfm infections. Reduced susceptibility to β-lactams in *E. faecium*, predominantly within the clade A1,^15,16^ has been associated with the overexpression of the low-affinity Pbp5^17,18^ and, more recently, to other PBPs such as PbpF, PonA, or PbpA.^19,20^ However, approximately 7.5% of *E. faecium* clinical isolates in our region remain susceptible to ampicillin.^21^ While our experiments used standardized *in vitro* conditions (e.g. fixed antibiotic concentrations and defined media), our primary aim was to assess the synergistic potential of ampicillin combined with cephalosporins under controlled experimental parameters, as done in previous studies evaluating β-lactam synergy.^8,22^

Some ASEfm strains under study exhibited low MICs for ceftaroline, ceftriaxone, and cefotaxime. This observation is noteworthy because *E. faecium* has traditionally been considered intrinsically resistant to all cephalosporins.^23^ Cephalosporin resistance in enterococci has been associated with the reduced binding affinity of these antibiotics to the Pbp5 protein, a key PBP. Additionally, previous studies have shown that mutants in the CroRS two-component regulatory system or the class B penicillin-binding protein PbpA are CPH-S.^20,24^ Importantly, all these mutant strains were genetically engineered in the laboratory, making our current finding of naturally occurring CPH-S clinical isolates particularly novel. Further work is needed to determine the genetic causes of the CPH-S phenotype in our clinical isolates.

While ampicillin plus ceftriaxone is a first-line therapy for *E. faecalis* endocarditis,^6,7^ data on its efficacy against ASEfm infections is limited. Synergism between amoxicillin and cefotaxime against *E. faecium* strains has been reported inconsistently.^8^ A recent study found synergism between ampicillin and ceftriaxone in only 3 out of 9 (33.3%) ASEfm isolates, showing MICs for ampicillin between 0.25 and 0.5 mg/L.^8^ These findings are in line with our results and reinforce the notion that the synergistic effect of ampicillin combined with ceftriaxone against ASEfm is variable across strains. While our results do not support a consistent correlation between low ampicillin MICs and synergism—since some strains with MICs ranging from 0.064 to 0.75 mg/L did not exhibit synergism, this variability appears consistent with previous studies. This suggests that MIC alone may not predict the presence or absence of synergism between ampicillin and cephalosporins. Thus, it may be recommendable to perform specific assays to assess potential synergism between these antibiotics, should they be considered a therapeutic option for a difficult-to-treat infection caused by this pathogen.

Previous studies have demonstrated that ampicillin plus ceftaroline exhibits synergistic effects against *E. faecalis*, which is susceptible to ampicillin.^22^ This synergy is also well-documented in *E. faecium* strains that are typically resistant to ampicillin (AREfm). Our research found significant synergistic effects of ampicillin and ceftaroline against nearly half of the *E. faecium* isolates studied, which are ampicillin-susceptible. This finding suggests that the effectiveness of the ampicillin-ceftaroline combination may extend beyond strains with established ampicillin resistance, supporting its potential as a therapeutic option for infections caused by ASEfm.

Importantly, the combinations tested in our assays at MIC concentrations did not display bactericidal activity against ASEfm, with the exception of one strain. This lack of widespread bactericidal effect, despite observed synergistic or additive interactions, may be attributed to several factors. Firstly, the MIC concentrations used in the assays might be insufficient for achieving complete bactericidal eradication. The concentrations required for bactericidal activity often exceed those needed merely to inhibit bacterial growth. Additionally, the strains possess mechanisms of resistance or adaptive responses that allow survival even in the presence of effective antibiotic combinations. The effectiveness of the combinations at higher concentrations, which more accurately simulate physiological conditions, remains unexplored. In the same line, the use of *in vitro* models simulating human pharmacokinetics could provide further insights into the clinical effectiveness of β-lactams at concentrations higher than the MIC. Although the precise mechanisms underlying the strain-dependent differences remain unclear, we speculate that intrinsic variability in cell wall synthesis, or its regulation might influence the observed responses to β-lactam combinations. These intrinsic factors could include differential expression or activity of PBPs,^25^ the presence of clade-specific *psr* sequences,^26^ variations in regulatory networks controlling cell wall homeostasis,^27,28^ or yet unidentified resistance mechanisms. Moreover, while our study was not designed to investigate the distribution of different PBPs in the studied strains, this limitation, along with the relatively small number of strains analyzed, restricts the generalizability of our results. Despite these constraints, our findings suggest that ampicillin and ceftaroline could exhibit synergistic activity against ASEfm strains and may represent a viable therapeutic option for challenging difficult-to-treat infections caused by ASEfm. Future studies, including *in vivo* validation using animal models, will be necessary to confirm and expand upon our findings.

## Supplementary Material

dkaf226_Supplementary_Data

## Supplementary data

Tables S1 to S3 is available as Supplementary data at *JAC*  Online.
