# Supplementary material for: Is ampicillin plus cephalosporins a therapeutic option for Ampicillin-Susceptible Enterococcus faecium?
Source: J Antimicrob Chemother. 2025 Aug 6;80(10):2622–9. doi: 10.1093/jac/dkaf226 (PMC12494129; doi:10.1093/jac/dkaf226)
Supplement: dkaf226_Supplementary_Data [file dkaf226_supplementary_data.zip › Table S1.docx]

| **MIC (mg/L)** | | | | |
| --- | --- | --- | --- | --- |
| **Strain** | **AMP** | **CTR** | **CTL** | **VAN** |
| Efm-1 | 0.19 | 4 | 0.25 | 1 |
| Efm-2 | 0.75 | 48 | 0.38 | <=1 |
| Efm-3 | 2 | >256 | 0.75 | <=1 |
| Efm-4 | 0.5 | 256 | 0.38 | 2 |
| Efm-5 | 0.25 | 3 | 0.19 | 4 |
| Efm-6 | 1 | >256 | 0.38 | 1 |
| Efm-9 | 0.75 | 12 | 0.25 | 4 |
| Efm-10 | 0.75 | 3 | 0.25 | 4 |
| Efm-54 | 0.064 | 1 | 0.064 | 1 |
| Efm-57 | 4 | >256 | 0.5 | 1 |

**B**

**A**

| **MIC (mg/L)** | | | | |
| --- | --- | --- | --- | --- |
| **Strain** | **AMP** | **CTR** | **CTL** | **VAN** |
| Efm-1 | 0,25 | 4 | 0,25 | 0,5 |
| Efm-2 | 1 | 32 | 0,5 | 0,5 |
| Efm-3 | 4 | >256 | 1 | 0,25 |
| Efm-4 | 1 | 32 | 0,5 | 2 |
| Efm-5 | 0,5 | 4 | 0,12 | 2 |
| Efm-6 | 1 | >256 | 0,5 | 0,5 |
| Efm-9 | 1 | 8 | 0,5 | 0,5 |
| Efm-10 | 1 | 2 | 0,5 | 0,25 |
| Efm-54 | 1 | 16 | 0,5 | 2 |
| Efm-57 | 2 | >256 | 1 | 0,25 |
|  |  |  |  |  |
| ATCC 29213 (*S.aureus*) | 0,5 | 4 | 0,25 | 0,5 |
| ATCC 29212 (*E faecalis*) | 1 | 8 | 0,5 | 2 |
