# Supplementary material for: Is ampicillin plus cephalosporins a therapeutic option for Ampicillin-Susceptible Enterococcus faecium?
Source: J Antimicrob Chemother. 2025 Aug 6;80(10):2622–9. doi: 10.1093/jac/dkaf226 (PMC12494129; doi:10.1093/jac/dkaf226)
Supplement: dkaf226_Supplementary_Data [file dkaf226_supplementary_data.zip › Table S2.docx]

| **Ampicillin + ceftaroline** | | | | |
| --- | --- | --- | --- | --- |
|  |  | log10 CFU/ml | ΔChange (log10 CFU/ml) |  |
|  | **Conditions** | **0h** | **4h 24h** | **AMP+CTL** |
|  | Blank | 5,8 | +1,5 +1,6 | AMP1/2xMIC+CTL1/2xMIC Indifference |
|  | AMP1/2xMIC | 5,8 | +1,1 +1,2 | AMP1xMIC+CTL1xMIC Additive |
|  | AMP1xMIC | 5,8 | +0,8 +1,0 | AMP1/2xMIC+CTL1xMIC Additive |
| Efm1 | CTL1/2xMIC  CTL1xMIC | 5,8  5,8 | +0,6 +0,7  +0,3 +0,3 | AMP1xMIC+CTL1/2xMIC Additive |
|  |  |  |  |  |
|  | AMP1/2xMIC+CTL1/2xMIC | 5,8 | +0,3 +0,1 |  |
|  | AMP1xMIC+CTL1xMIC | 5,8 | +0,3 -1,2 |  |
|  | AMP1/2xMIC+CTL1xMIC | 5,8 | -0,1 -0,9 |  |
|  | AMP1xMIC+CTL1/2xMIC | 5,8 | +0,0 -0,9 |  |
|  | **Conditions** | **0h** | **4h 24h** | **AMP+CTL** |
|  | Blank | 5,7 | +1,7 +1,8 | AMP1/2xMIC+CTL1/2xMIC Indifference |
|  | AMP1/2xMIC | 5,7 | +1,5 +1,6 | AMP1xMIC+CTL1xMIC Synergy |
|  | AMP1xMIC | 5,7 | +1,4 +1,4 | AMP1/2xMIC+CTL1xMIC Synergy |
| Efm2 | CTL1/2xMIC  CTL1xMIC | 5,7  5,7 | +1,0 +1,2  +0,8 +0,9 | AMP1xMIC+CTL1/2xMIC Additive |
|  |  |  |  |  |
|  | AMP1/2xMIC+CTL1/2xMIC | 5,7 | +0,6 +0,7 |  |
|  | AMP1xMIC+CTL1xMIC | 5,7 | -0,6 -1,7 |  |
|  | AMP1/2xMIC+CTL1xMIC | 5,7 | -0,3 -1,4 |  |
|  | AMP1xMIC+CTL1/2xMIC | 5,7 | +0,1 -0,8 |  |
|  | **Conditions** | **0h** | **4h 24h** | **AMP+CTL** |
|  | Blank | 5,8 | +1,7 +1,8 | AMP1/2xMIC+CTL1/2xMIC Indifference |
|  | AMP1/2xMIC | 5,8 | +1,6 +1,6 | AMP1xMIC+CTL1xMIC Synergy |
|  | AMP1xMIC | 5,8 | +1,4 +1,6 | AMP1/2xMIC+CTL1xMIC Additive |
| Efm3 | CTL1/2xMIC  CTL1xMIC | 5,7  5,8 | +1,3 +1,4  +1,0 +1,0 | AMP1xMIC+CTL1/2xMIC Indifference |
|  |  |  |  |  |
|  | AMP1/2xMIC+CTL1/2xMIC | 5,7 | +1,0 +1,0 |  |
|  | AMP1xMIC+CTL1xMIC | 5,7 | -0,6 -1,8 |  |
|  | AMP1/2xMIC+CTL1xMIC | 5,7 | -0,3 -0,8 |  |
|  | AMP1xMIC+CTL1/2xMIC | 5,7 | +0,8 +0,5 |  |
|  | **Conditions** | **0h** | **4h 24h** | **AMP+CTL** |
|  | Blank | 5,8 | +1,7 +1,8 | AMP1/2xMIC+CTL1/2xMIC Indifference |
|  | AMP1/2xMIC | 5,8 | +1,5 +1,5 | AMP1xMIC+CTL1xMIC Synergy |
|  | AMP1xMIC | 5,8 | +1,2 +1,4 | AMP1/2xMIC+CTL1xMIC Additive |
| Efm4 | CTL1/2xMIC  CTL1xMIC | 5,8  5,8 | +1,1 +1,0  +0,5 +0,5 | AMP1xMIC+CTL1/2xMIC Additive |
|  |  |  |  |  |
|  | AMP1/2xMIC+CTL1/2xMIC | 5,8 | +0,4 +0,4 |  |
|  | AMP1xMIC+CTL1xMIC | 5,8 | -0,5 -1,9 |  |
|  | AMP1/2xMIC+CTL1xMIC | 5,8 | -0,2 -1,4 |  |
|  | AMP1xMIC+CTL1/2xMIC | 5,8 | +0,2 -0,7 |  |
|  | **Conditions** | **0h** | **4h 24h** | **AMP+CTL** |
|  | Blank | 5,9 | +1,6 +1,6 | AMP1/2xMIC+CTL1/2xMIC Indifference |
|  | AMP1/2xMIC | 5,9 | +1,3 +1,3 | AMP1xMIC+CTL1xMIC Additive |
|  | AMP1xMIC | 5,9 | +1,0 +1,3 | AMP1/2xMIC+CTL1xMIC Indifference |
| Efm5 | CTL1/2xMIC  CTL1xMIC | 5,9  5,9 | +1,0 +1,0  +0,7 +0,8 | AMP1xMIC+CTL1/2xMIC Indifference |
|  |  |  |  |  |
|  | AMP1/2xMIC+CTL1/2xMIC | 5,9 | +0,7 +0,7 |  |
|  | AMP1xMIC+CTL1xMIC | 5,9 | +0,2 -0,3 |  |
|  | AMP1/2xMIC+CTL1xMIC | 5,9 | +0,3 +0,4 |  |
|  | AMP1xMIC+CTL1/2xMIC | 5,9 | +0,5 +0,5 |  |

|  | **Conditions** | **0h** | **4h** | **24h** | **AMP+CTL** |
| --- | --- | --- | --- | --- | --- |
|  | Blank | 6,0 | +1,3 | +1,4 | AMP1/2xMIC+CTL1/2xMIC Indifference |
|  | AMP1/2xMIC | 6,0 | +0,7 | +1,1 | AMP1xMIC+CTL1xMIC Synergy |
|  | AMP1xMIC | 6,0 | +0,4 | +0,6 | AMP1/2xMIC+CTL1xMIC Synergy |
| Efm6 | CTL1/2xMIC  CTL1xMIC | 6,0  6,0 | +0,8  +0,1 | +1,1  +0,6 | AMP1xMIC+CTL1/2xMIC Synergy |
|  | AMP1/2xMIC+CTL1/2xMIC | 6,0 | +0,2 | +0,4 |  |
|  | AMP1xMIC+CTL1xMIC | 6,0 | -0,5 | -2,4 |  |
|  | AMP1/2xMIC+CTL1xMIC | 6,0 | -0,4 | -2,5 |  |
|  | AMP1xMIC+CTL1/2xMIC | 6,0 | -0,2 | -1,7 |  |
|  | **Conditions** | **0h** | **4h** | **24h** | **AMP+CTL** |
|  | Blank | 5,9 | +1,5 | +1,6 | AMP1/2xMIC+CTL1/2xMIC Indifference |
|  | AMP1/2xMIC | 5,9 | +1,3 | +1,4 | AMP1xMIC+CTL1xMIC Additive |
|  | AMP1xMIC | 5,9 | +1,2 | +1,2 | AMP1/2xMIC+CTL1xMIC Indifference |
| Efm9 | CTL1/2xMIC  CTL1xMIC | 5,9  5,9 | +1,2  +1,1 | +1,2  +1,1 | AMP1xMIC+CTL1/2xMIC Indifference |
|  | AMP1/2xMIC+CTL1/2xMIC | 5,9 | +0,9 | +1,0 |  |
|  | AMP1xMIC+CTL1xMIC | 5,9 | +0,2 | +0,0 |  |
|  | AMP1/2xMIC+CTL1xMIC | 5,9 | +0,7 | +0,6 |  |
|  | AMP1xMIC+CTL1/2xMIC | 5,9 | +0,6 | +0,7 |  |
|  | **Conditions** | **0h** | **4h** | **24h** | **AMP+CTL** |
|  | Blank | 5,7 | +1,5 | +1,9 | AMP1/2xMIC+CTL1/2xMIC Indifference |
|  | AMP1/2xMIC | 5,7 | +1,2 | +1,6 | AMP1xMIC+CTL1xMIC Indifference |
|  | AMP1xMIC | 5,7 | +1,1 | +1,5 | AMP1/2xMIC+CTL1xMIC Indifference |
| Efm10 | CTL1/2xMIC  CTL1xMIC | 5,7  5,7 | +1,1  +0,8 | +1,5  +1,3 | AMP1xMIC+CTL1/2xMIC Indifference |
|  | AMP1/2xMIC+CTL1/2xMIC | 5,7 | +0,9 | +1,4 |  |
|  | AMP1xMIC+CTL1xMIC | 5,7 | +0,6 | +0,9 |  |
|  | AMP1/2xMIC+CTL1xMIC | 5,7 | +0,5 | +0,9 |  |
|  | AMP1xMIC+CTL1/2xMIC | 5,7 | +0,8 | +1,3 |  |
|  | **Conditions** | **0h** | **4h** | **24h** | **AMP+CTL** |
|  | Blank | 5,6 | +1,6 | +1,6 | AMP1/2xMIC+CTL1/2xMIC Indifference |
|  | AMP1/2xMIC | 5,6 | +1,3 | +1,4 | AMP1xMIC+CTL1xMIC Indifference |
|  | AMP1xMIC | 5,6 | +1,2 | +1,1 | AMP1/2xMIC+CTL1xMIC Indifference |
| Efm54 | CTL1/2xMIC  CTL1xMIC | 5,6  5,6 | +1,1  +0,8 | +1,1  +1,0 | AMP1xMIC+CTL1/2xMIC Indifference |
|  | AMP1/2xMIC+CTL1/2xMIC | 5,6 | +1,1 | +1,0 |  |
|  | AMP1xMIC+CTL1xMIC | 5,6 | +0,6 | +0,6 |  |
|  | AMP1/2xMIC+CTL1xMIC | 5,6 | +0,8 | +0,8 |  |
|  | AMP1xMIC+CTL1/2xMIC | 5,6 | +0,8 | +0,9 |  |
|  | **Conditions** | **0h** | **4h** | **24h** | **AMP+CTL** |
|  | Blank | 5,5 | +1,8 | +1,8 | AMP1/2xMIC+CTL1/2xMIC Indifference |
|  | AMP1/2xMIC | 5,5 | +1,5 | +1,6 | AMP1xMIC+CTL1xMIC Indifference |
|  | AMP1xMIC | 5,5 | +1,5 | +1,6 | AMP1/2xMIC+CTL1xMIC Indifference |
| Efm57 | CTL1/2xMIC  CTL1xMIC | 5,5  5,5 | +1,8  +1,9 | +1,9  +1,8 | AMP1xMIC+CTL1/2xMIC Indifference |
|  | AMP1/2xMIC+CTL1/2xMIC | 5,5 | +1,5 | +1,6 |  |
|  | AMP1xMIC+CTL1xMIC | 5,5 | +1,3 | +1,1 |  |
|  | AMP1/2xMIC+CTL1xMIC | 5,5 | +1,5 | +1,3 |  |
|  | AMP1xMIC+CTL1/2xMIC | 5,5 | +1,2 | +1,2 |  |

| **Ampicillin + ceftriaxone** | | | | | |
| --- | --- | --- | --- | --- | --- |
|  |  | log10 CFU/ml | ΔChange (log10 | CFU/ml) |  |
|  | **Conditions** | **0h** | **4h** | **24h** | **AMP+CTR** |
|  | Blank | 5,8 | +1,5 | +1,5 | AMP1/2xMIC+CTR1/2xMIC Indifference |
|  | AMP1/2xMIC | 5,8 | +0,9 | +1,0 | AMP1xMIC+CTR1xMIC Indifference |
|  | AMP1xMIC | 5,8 | +0,7 | +0,7 | AMP1/2xMIC+CTR1xMIC Indifference |
| Efm1 | CTR1/2xMIC  CTR1xMIC | 5,8  5,8 | +0,1  +0,1 | -0,9  -1,0 | AMP1xMIC+CTR1/2xMIC Indifference |
|  | AMP1/2xMIC+CTR1/2xMIC | 5,8 | +0,1 | -0,9 |  |
|  | AMP1xMIC+CTR1xMIC | 5,9 | -0,2 | -1,2 |  |
|  | AMP1/2xMIC+CTR1xMIC | 5,8 | -0,0 | -1,5 |  |
|  | AMP1xMIC+CTR1/2xMIC | 5,8 | -0,1 | -1,3 |  |
|  | **Conditions** | **0h** | **4h** | **24h** | **AMP+CTR** |
|  | Blank | 5,9 | +1,5 | +1,6 | AMP1/2xMIC+CTR1/2xMIC Indifference |
|  | AMP1/2xMIC | 5,8 | +1,3 | +1,4 | AMP1xMIC+CTR1xMIC Indifference |
|  | AMP1xMIC | 5,8 | +1,0 | +0,8 | AMP1/2xMIC+CTR1xMIC Indifference |
| Efm2 | CTR1/2xMIC  CTR1xMIC | 5,9  5,9 | +0,3  +0,2 | -0,0  -0,4 | AMP1xMIC+CTR1/2xMIC Additive |
|  | AMP1/2xMIC+CTR1/2xMIC | 5,8 | +0,1 | -0,4 |  |
|  | AMP1xMIC+CTR1xMIC | 5,9 | -0,3 | -1,1 |  |
|  | AMP1/2xMIC+CTR1xMIC | 5,9 | -0,4 | -0,4 |  |
|  | AMP1xMIC+CTR1/2xMIC | 5,9 | +0,9 | +1,0 |  |
|  | **Conditions** | **0h** | **4h** | **24h** | **AMP+CTR** |
|  | Blank | 5,7 | +1,8 | +1,8 | AMP1/2xMIC+CTRCmax/2 Indifference |
|  | AMP1/2xMIC | 5,7 | +1,7 | +1,8 | AMP1xMIC+CTRCmax Indifference |
|  | AMP1xMIC | 5,7 | +1,6 | +1,6 | AMP1/2xMIC+CTRCmax Indifference |
| Efm3 | CTRCmax/2  CTRCmax/2 | 5,7  5,7 | +1,0  +0,9 | +0,9  +0,7 | AMP1xMIC+CTRCmax/2 Indifference |
|  | AMP1/2xMIC+CTRCmax/2 | 5,7 | +1,2 | +1,1 |  |
|  | AMP1xMIC+CTRCmax | 5,7 | +0,9 | +0,8 |  |
|  | AMP1/2xMIC+CTRCmax | 5,7 | +0,8 | +0,4 |  |
|  | AMP1xMIC+CTRCmax/2 | 5,7 | +1,0 | +0,9 |  |
|  | **Conditions** | **0h** | **4h** | **24h** | **AMP+CTR** |
|  | Blank | 5,9 | +1,7 | +1,6 | AMP1/2xMIC+CTRCmax/2 Indifference |
|  | AMP1/2xMIC | 5,9 | +1,4 | +1,5 | AMP1xMIC+CTRCmax Synergy |
|  | AMP1xMIC | 5,9 | +1,1 | +1,3 | AMP1/2xMIC+CTRCmax Synergy |
| Efm4 | CTRCmax/2  CTRCmax/2 | 5,9  5,9 | -0,0  +0,5 | +0,5  +0,2 | AMP1xMIC+CTRCmax/2 Additive |
|  | AMP1/2xMIC+CTRCmax/2 | 5,9 | -0,3 | -0,4 |  |
|  | AMP1xMIC+CTRCmax | 5,9 | -0,9 | -2,1 |  |
|  | AMP1/2xMIC+CTRCmax | 5,9 | -0,6 | -2,0 |  |
|  | AMP1xMIC+CTRCmax/2 | 5,9 | -0,5 | -0,6 |  |
|  | **Conditions** | **0h** | **4h** | **24h** | **AMP+CTR** |
|  | Blank | 5,9 | +1,6 | +1,6 | AMP1/2xMIC+CTR1/2xMIC Indifference |
|  | AMP1/2xMIC | 5,9 | +1,4 | +1,3 | AMP1xMIC+CTR1xMIC Indifference |
|  | AMP1xMIC | 5,9 | +1,2 | +1,1 | AMP1/2xMIC+CTR1xMIC Indifference |
| Efm5 | CTR1/2xMIC  CTR1xMIC | 5,9  5,9 | +0,5  +0,3 | +0,2  +0,1 | AMP1xMIC+CTR1/2xMIC Indifference |
|  | AMP1/2xMIC+CTR1/2xMIC | 5,9 | +0,6 | +0,5 |  |
|  | AMP1xMIC+CTR1xMIC | 5,9 | +0,4 | +0,2 |  |
|  | AMP1/2xMIC+CTR1xMIC | 5,9 | +0,3 | +0,3 |  |
|  | AMP1xMIC+CTR1/2xMIC | 5,9 | +0,7 | +0,7 |  |

|  | **Conditions** | **0h** | **4h** | **24h** | **AMP+CTR** |
| --- | --- | --- | --- | --- | --- |
|  | Blank | 6,0 | +1,2 | +1,3 | AMP1/2xMIC+CTRCmax/2 Indifference |
|  | AMP1/2xMIC | 6,0 | +0,6 | +1,1 | AMP1xMIC+CTRCmax Synergy |
|  | AMP1xMIC | 6,0 | +0,3 | +1,0 | AMP1/2xMIC+CTRCmax Synergy |
| Efm6 | CTRCmax/2  CTRCmax/2 | 6,0  6,0 | +0,4  +0,3 | +0,4  +0,3 | AMP1xMIC+CTRCmax/2 Synergy |
|  | AMP1/2xMIC+CTRCmax/2 | 6,0 | -0,2 | -0,6 |  |
|  | AMP1xMIC+CTRCmax | 6,0 | -0,5 | -3,3 |  |
|  | AMP1/2xMIC+CTRCmax | 6,0 | -0,3 | -2,6 |  |
|  | AMP1xMIC+CTRCmax/2 | 6,0 | -0,2 | -1,7 |  |
|  | **Conditions** | **0h** | **4h** | **24h** | **AMP+CTR** |
|  | Blank | 5,9 | +1,4 | +1,6 | AMP1/2xMIC+CTR1/2xMIC Indifference |
|  | AMP1/2xMIC | 5,9 | +1,3 | +1,3 | AMP1xMIC+CTR1xMIC Indifference |
|  | AMP1xMIC | 5,9 | +1,1 | +1,2 | AMP1/2xMIC+CTR1xMIC Indifference |
| Efm9 | CTR1/2xMIC  CTR1xMIC | 5,9  5,9 | +0,6  +0,5 | +0,4  +0,2 | AMP1xMIC+CTR1/2xMIC Indifference |
|  | AMP1/2xMIC+CTR1/2xMIC | 5,9 | +0,8 | +0,9 |  |
|  | AMP1xMIC+CTR1xMIC | 5,9 | +0,7 | +0,7 |  |
|  | AMP1/2xMIC+CTR1xMIC | 5,9 | +0,7 | +0,6 |  |
|  | AMP1xMIC+CTR1/2xMIC | 5,9 | +0,9 | +1,0 |  |
|  | **Conditions** | **0h** | **4h** | **24h** | **AMP+CTR** |
|  | Blank | 5,7 | +1,4 | +1,8 | AMP1/2xMIC+CTR1/2xMIC Indifference |
|  | AMP1/2xMIC | 5,7 | +1,3 | +1,6 | AMP1xMIC+CTR1xMIC Indifference |
|  | AMP1xMIC | 5,7 | +1,1 | +1,5 | AMP1/2xMIC+CTR1xMIC Indifference |
| Efm10 | CTR1/2xMIC  CTR1xMIC | 5,7  5,7 | +0,4  +0,3 | +0,4  +0,4 | AMP1xMIC+CTR1/2xMIC Indifference |
|  | AMP1/2xMIC+CTR1/2xMIC | 5,7 | +0,8 | +1,2 |  |
|  | AMP1xMIC+CTR1xMIC | 5,7 | +0,8 | +1,1 |  |
|  | AMP1/2xMIC+CTR1xMIC | 5,7 | +0,8 | +1,0 |  |
|  | AMP1xMIC+CTR1/2xMIC | 5,7 | +1,0 | +1,4 |  |
|  | **Conditions** | **0h** | **4h** | **24h** | **AMP+CTR** |
|  | Blank | 5,6 | +1,7 | +1,7 | AMP1/2xMIC+CTR1/2xMIC Indifference |
|  | AMP1/2xMIC | 5,6 | +1,5 | +1,4 | AMP1xMIC+CTR1xMIC Indifference |
|  | AMP1xMIC | 5,6 | +1,1 | +1,3 | AMP1/2xMIC+CTR1xMIC Indifference |
| Efm54 | CTR1/2xMIC  CTR1xMIC | 5,6  5,6 | +0,6  -0,1 | +0,3  -0,5 | AMP1xMIC+CTR1/2xMIC Indifference |
|  | AMP1/2xMIC+CTR1/2xMIC | 5,6 | +0,2 | +0,5 |  |
|  | AMP1xMIC+CTR1xMIC | 5,6 | +0,3 | +0,3 |  |
|  | AMP1/2xMIC+CTR1xMIC | 5,6 | +0,2 | +0,3 |  |
|  | AMP1xMIC+CTR1/2xMIC | 5,6 | +0,9 | +0,9 |  |
|  | **Conditions** | **0h** | **4h** | **24h** | **AMP+CTR** |
|  | Blank | 5,5 | +1,7 | +1,8 | AMP1/2xMIC+CTRCmax/2 Indifference |
|  | AMP1/2xMIC | 5,5 | +1,4 | +1,6 | AMP1xMIC+CTRCmax Indifference |
|  | AMP1xMIC | 5,5 | +1,3 | +1,4 | AMP1/2xMIC+CTRCmax Indifference |
| Efm57 | CTRCmax/2  CTRCmax/2 | 5,5  5,5 | +1,5  +1,5 | +1,6  +1,6 | AMP1xMIC+CTRCmax/2 Indifference |
|  | AMP1/2xMIC+CTRCmax/2 | 5,5 | +1,3 | +1,2 |  |
|  | AMP1xMIC+CTRCmax | 5,5 | +0,7 | +0,6 |  |
|  | AMP1/2xMIC+CTRCmax | 5,5 | +1,1 | +0,8 |  |
|  | AMP1xMIC+CTRCmax/2 | 5,5 | +0,8 | +0,8 |  |
