# Supplementary material for: Is ampicillin plus cephalosporins a therapeutic option for Ampicillin-Susceptible Enterococcus faecium?
Source: J Antimicrob Chemother. 2025 Aug 6;80(10):2622–9. doi: 10.1093/jac/dkaf226 (PMC12494129; doi:10.1093/jac/dkaf226)
Supplement: dkaf226_Supplementary_Data [file dkaf226_supplementary_data.zip › Table S3.docx]

| **AMP + CTL** | | | |
| --- | --- | --- | --- |
| **Condition** | **Mean_log10_24h** | **SD_log10_24h** | |
| **EFm1** | | | |
| Blank | 7,44 | 0,17 | |
| AMP ½ x MIC | 6,86 | 0,48 | |
| AMP 1 x MIC | 6,60 | 0,61 | |
| CTL ½ x MIC | 6,08 | 0,86 | |
| CTL 1 x MIC | 5,57 | 0,94 | |
| AMP 1 x MIC + CTL ½ x MIC | 4,72 | 0,52 | |
| AMP 1 x MIC + CTL 1 x MIC | 4,59 | 0,31 | |
| AMP ½ x MIC + CTL 1 MIC | 4,83 | 0,28 | |
| AMP ½ x MIC + CTL ½ x MIC | 5,45 | 0,84 | |
| **EFm2** | | | |
| Blank | 7,47 | 0,03 | |
| AMP ½ x MIC | 7,30 | 0,06 | |
| AMP 1 x MIC | 7,13 | 0,07 | |
| CTL ½ x MIC | 6,91 | 0,11 | |
| CTL 1 x MIC | 6,59 | 0,30 | |
| AMP 1 x MIC + CTL ½ x MIC | 4,55 | 0,74 | |
| AMP 1 x MIC + CTL 1 x MIC | 3,93 | 0,37 | |
| AMP ½ x MIC + CTL 1 MIC | 4,21 | 0,34 | |
| AMP ½ x MIC + CTL ½ x MIC | 6,40 | 0,17 | |
| **EFm3** | | | |
| Blank | 7,53 | 0,07 | |
| AMP ½ x MIC | 7,34 | 0,12 | |
| AMP 1 x MIC | 7,31 | 0,13 | |
| CTL ½ x MIC | 7,09 | 0,28 | |
| CTL 1 x MIC | 6,63 | 0,36 | |
| AMP 1 x MIC + CTL ½ x MIC | 6,21 | 0,33 | |
| AMP 1 x MIC + CTL 1 x MIC | 3,84 | 0,26 | |
| AMP ½ x MIC + CTL 1 MIC | 4,36 | 0,86 | |
| AMP ½ x MIC + CTL ½ x MIC | 6,61 | 0,37 | |
| **EFm4** | | | |
| Blank | 7,57 | 0,04 | |
| AMP ½ x MIC | 7,31 | 0,16 | |
| AMP 1 x MIC | 7,21 | 0,07 | |
| CTL ½ x MIC | 6,83 | 0,11 | |
| CTL 1 x MIC | 6,27 | 0,04 | |
| AMP 1 x MIC + CTL ½ x MIC | 5,17 | 0,05 | |
| AMP 1 x MIC + CTL 1 x MIC | 3,90 | 0,04 | |
| AMP ½ x MIC + CTL 1 MIC | 4,37 | 0,31 | |
| AMP ½ x MIC + CTL ½ x MIC | 6,28 | 0,01 | |
| **EFm5** | | | |
| Blank | 7,50 | 0,00 | |
| AMP ½ x MIC | 7,29 | 0,00 | |
| AMP 1 x MIC | 7,21 | 0,14 | |
| CTL ½ x MIC | 6,88 | 0,05 | |
| CTL 1 x MIC | 6,76 | 0,07 | |
| AMP 1 x MIC + CTL ½ x MIC | 6,45 | 0,10 | |
| AMP 1 x MIC + CTL 1 x MIC | 5,59 | 0,07 | |
| AMP ½ x MIC + CTL 1 MIC | 6,28 | 0,19 | |
| AMP ½ x MIC + CTL ½ x MIC | 6,68 | 0,20 | |
| **EFm6** | | | |
| Blank | 7,37 | | 0,15 |
| AMP ½ x MIC | 7,08 | | 0,15 |
| AMP 1 x MIC | 6,63 | | 0,11 |
| CTL ½ x MIC | 7,15 | | 0,12 |
| CTL 1 x MIC | 6,52 | | 0,37 |
| AMP 1 x MIC + CTL ½ x MIC | 4,07 | | 0,74 |
| AMP 1 x MIC + CTL 1 x MIC | 3,54 | | 0,34 |
| AMP ½ x MIC + CTL 1 MIC | 3,54 | | 0,00 |
| AMP ½ x MIC + CTL ½ x MIC | 6,28 | | 0,45 |
| **EFm9** | | | |
| Blank | 7,46 | | 0,09 |
| AMP ½ x MIC | 7,15 | | 0,34 |
| AMP 1 x MIC | 7,03 | | 0,31 |
| CTL ½ x MIC | 7,00 | | 0,42 |
| CTL 1 x MIC | 6,60 | | 0,72 |
| AMP 1 x MIC + CTL ½ x MIC | 6,37 | | 0,56 |
| AMP 1 x MIC + CTL 1 x MIC | 5,60 | | 0,54 |
| AMP ½ x MIC + CTL 1 MIC | 6,16 | | 0,59 |
| AMP ½ x MIC + CTL ½ x MIC | 6,67 | | 0,50 |
| **EFm10** | | | |
| Blank | 7,51 | | 0,11 |
| AMP ½ x MIC | 7,17 | | 0,37 |
| AMP 1 x MIC | 7,13 | | 0,28 |
| CTL ½ x MIC | 7,00 | | 0,68 |
| CTL 1 x MIC | 6,80 | | 0,67 |
| AMP 1 x MIC + CTL ½ x MIC | 6,96 | | 0,13 |
| AMP 1 x MIC + CTL 1 x MIC | 6,43 | | 0,40 |
| AMP ½ x MIC + CTL 1 MIC | 6,44 | | 0,49 |
| AMP ½ x MIC + CTL ½ x MIC | 6,93 | | 0,43 |
| **EFm54** | | | |
| Blank | 7,23 | | 0,09 |
| AMP ½ x MIC | 6,97 | | 0,21 |
| AMP 1 x MIC | 6,76 | | 0,03 |
| CTL ½ x MIC | 6,77 | | 0,02 |
| CTL 1 x MIC | 6,59 | | 0,08 |
| AMP 1 x MIC + CTL ½ x MIC | 6,52 | | 0,05 |
| AMP 1 x MIC + CTL 1 x MIC | 6,26 | | 0,18 |
| AMP ½ x MIC + CTL 1 MIC | 6,43 | | 0,00 |
| AMP ½ x MIC + CTL ½ x MIC | 6,66 | | 0,09 |
| **EFm57** | | | |
| Blank | 7,37 | | 0,05 |
| AMP ½ x MIC | 7,14 | | 0,10 |
| AMP 1 x MIC | 7,15 | | 0,09 |
| CTL ½ x MIC | 7,38 | | 0,05 |
| CTL 1 x MIC | 7,34 | | 0,12 |
| AMP 1 x MIC + CTL ½ x MIC | 6,73 | | 0,11 |
| AMP 1 x MIC + CTL 1 x MIC | 6,61 | | 0,01 |
| AMP ½ x MIC + CTL 1 MIC | 6,79 | | 0,02 |
| AMP ½ x MIC + CTL ½ x MIC | 7,09 | | 0,12 |

| **AMP + CTR** | | |
| --- | --- | --- |
| **Condition** | **log10_24h** | **SD_log10_24h** |
| **EFm1** | | |
| Blank | 7,33 | 0,19 |
| AMP ½ x MIC | 6,72 | 0,40 |
| AMP 1 x MIC | 6,42 | 0,45 |
| CTR ½ x MIC | 4,69 | 0,49 |
| CTR 1 x MIC | 4,58 | 0,47 |
| AMP 1 x MIC + CTR ½ x MIC | 4,48 | 0,24 |
| AMP 1 x MIC + CTR 1 x MIC | 4,55 | 0,35 |
| AMP ½ x MIC + CTR 1 x MIC | 4,29 | 0,27 |
| AMP ½ x MIC + CTR ½ x MIC | 4,71 | 0,42 |
| **EFm2** | | |
| Blank | 7,39 | 0,25 |
| AMP ½ x MIC | 7,10 | 0,49 |
| AMP 1 x MIC | 6,50 | 0,91 |
| CTR 1 x Cmax | 5,34 | 1,35 |
| CTR ½ x Cmax | 5,36 | 0,63 |
| AMP 1 x MIC + CTR 1 x Cmax | 4,84 | 1,00 |
| AMP 1 + CTR ½ x Cmax | 4,29 | 0,74 |
| AMP ½ + CTR 1 x Cmax | 4,60 | 1,39 |
| AMP ½ + CTR ½ x Cmax | 4,99 | 1,13 |
| **EFm3** | | |
| Blank | 7,46 | 0,06 |
| AMP ½ x MIC | 7,43 | 0,05 |
| AMP 1 x MIC | 7,26 | 0,08 |
| CTR 1 x Cmax | 6,57 | 0,06 |
| CTR ½ x Cmax | 6,37 | 0,04 |
| AMP 1 x MIC + CTR 1 x Cmax | 6,59 | 0,06 |
| AMP 1 + CTR ½ x Cmax | 6,43 | 0,09 |
| AMP ½ + CTR 1 x Cmax | 5,98 | 0,46 |
| AMP ½ + CTR ½ x Cmax | 6,73 | 0,02 |
| **EFm4** | | |
| Blank | 7,45 | 0,10 |
| AMP ½ x MIC | 7,31 | 0,10 |
| AMP 1 x MIC | 7,19 | 0,07 |
| CTR 1 x Cmax | 6,33 | 0,18 |
| CTR ½ x Cmax | 5,92 | 0,42 |
| AMP 1 x MIC + CTR 1 x Cmax | 5,21 | 0,11 |
| AMP 1 + CTR ½ x Cmax | 3,76 | 0,03 |
| AMP ½ + CTR 1 x Cmax | 3,83 | 0,02 |
| AMP ½ + CTR ½ x Cmax | 5,44 | 0,22 |
| **EFm5** | | |
| Blank | 7,51 | 0,02 |
| AMP ½ x MIC | 7,25 | 0,15 |
| AMP 1 x MIC | 7,04 | 0,19 |
| CTR ½ x MIC | 6,17 | 0,01 |
| CTR 1 x MIC | 5,98 | 0,24 |
| AMP 1 x MIC + CTR ½ x MIC | 6,63 | 0,17 |
| AMP 1 x MIC + CTR 1 x MIC | 6,12 | 0,03 |
| AMP ½ x MIC + CTR 1 x MIC | 6,25 | 0,17 |
| AMP ½ x MIC + CTR ½ x MIC | 6,41 | 0,12 |
| **EFm6** | | |
| Blank | 7,34 | 0,10 |
| AMP ½ x MIC | 7,10 | 0,18 |
| AMP 1 x MIC | 6,90 | 0,41 |
| CTR 1 x Cmax | 6,38 | 0,05 |
| CTR ½ x Cmax | 6,44 | 0,12 |
| AMP 1 x MIC + CTR 1 x Cmax | 2,70 | 0,00 |
| AMP 1 + CTR ½ x Cmax | 4,31 | 0,04 |
| AMP ½ + CTR 1 x Cmax | 3,35 | 0,49 |
| AMP ½ + CTR ½ x Cmax | 5,45 | 0,14 |
| **EFm9** | | |
| Blank | 7,49 | 0,08 |
| AMP ½ x MIC | 7,13 | 0,28 |
| AMP 1 x MIC | 7,08 | 0,19 |
| CTR ½ x MIC | 6,06 | 0,70 |
| CTR 1 x MIC | 5,84 | 0,78 |
| AMP 1 x MIC + CTR ½ x MIC | 6,78 | 0,40 |
| AMP 1 x MIC + CTR 1 x MIC | 6,45 | 0,51 |
| AMP ½ x MIC + CTR 1 x MIC | 6,34 | 0,67 |
| AMP ½ x MIC + CTR ½ x MIC | 6,64 | 0,62 |
| **EFm10** | | |
| Blank | 7,38 | 0,30 |
| AMP ½ x MIC | 7,17 | 0,33 |
| AMP 1 x MIC | 7,06 | 0,41 |
| CTR ½ x MIC | 5,90 | 0,59 |
| CTR 1 x MIC | 5,89 | 0,56 |
| AMP 1 x MIC + CTR ½ x MIC | 6,91 | 0,44 |
| AMP 1 x MIC + CTR 1 x MIC | 6,66 | 0,42 |
| AMP ½ x MIC + CTR 1 x MIC | 6,51 | 0,51 |
| AMP ½ x MIC + CTR ½ x MIC | 6,74 | 0,53 |
| **EFm54** | | |
| Blank | 7,24 | 0,05 |
| AMP ½ x MIC | 7,00 | 0,00 |
| AMP 1 x MIC | 6,83 | 0,13 |
| CTR ½ x MIC | 5,67 | 0,72 |
| CTR 1 x MIC | 5,05 | 0,29 |
| AMP 1 x MIC + CTR ½ x MIC | 6,49 | 0,03 |
| AMP 1 x MIC + CTR 1 x MIC | 5,81 | 0,27 |
| AMP ½ x MIC + CTR 1 x MIC | 5,80 | 0,37 |
| AMP ½ x MIC + CTR ½ x MIC | 5,95 | 0,56 |
| **EFm57** | | |
| Blank | 7,15 | 0,44 |
| AMP ½ x MIC | 6,92 | 0,55 |
| AMP 1 x MIC | 6,61 | 0,77 |
| CTR 1 x Cmax | 6,74 | 0,94 |
| CTR ½ x Cmax | 6,68 | 0,98 |
| AMP 1 x MIC + CTR 1 x Cmax | 6,07 | 0,68 |
| AMP 1 + CTR ½ x Cmax | 5,24 | 1,45 |
| AMP ½ + CTR 1 x Cmax | 5,67 | 1,10 |
| AMP ½ + CTR ½ x Cmax | 6,07 | 1,00 |
